# Inter-rater reliability of a national acute stroke register

**DOI:** 10.1186/s13104-015-1556-3

**Published:** 2015-10-19

**Authors:** Torunn Varmdal, Hanne Ellekjær, Hild Fjærtoft, Bent Indredavik, Stian Lydersen, Kaare Harald Bonaa

**Affiliations:** Department of Public Health and General Practice, Norwegian University of Science and Technology, Postbox 8905, 7401 Trondheim, Norway; Stroke Unit, St. Olav’s University Hospital, Trondheim, Norway; Department of Medical Quality Registries, St. Olav’s University Hospital, Trondheim, Norway; Department of Neuroscience, Norwegian University of Science and Technology, Trondheim, Norway; Clinic for Heart Disease, St. Olav’s University Hospital, Trondheim, Norway; Department of Community Medicine, UiT The Arctic University of Norway, Tromsö, Norway; Regional Centre for Child and Youth Mental Health and Child Welfare Central Norway, Norwegian University of Science and Technology, Trondheim, Norway

**Keywords:** Inter-rater reliability, Quality registers, Data quality

## Abstract

**Background:**

Medical quality registers are useful sources of knowledge about diseases and the health services. However, there are challenges in obtaining valid and reliable data. This study aims to assess the reliability in a national medical quality register.

**Methods:**

We randomly selected 111 patients having had a stroke in 2012. An experienced stroke nurse completed the Norwegian Stroke Register paper forms for all 111 patients by review of the medical records. We then extracted all registered data on the same patients from the Norwegian Stroke Register and calculated Cohen’s kappa and Gwet’s AC_1_ with 95 % confidence intervals for 51 nominal variables and Cohen’s quadratic weighted kappa and Gwet’s AC_2_ for three ordinal variables. For two time variables, we calculated the Intraclass Correlation Coefficient.

**Results:**

Substantial to excellent reliability (kappa > 0.60/AC_1_ > 0.80) was observed for most variables related to past medical history, functional status, stroke subtype and discharge destination. Although excellent reliability was observed for time of stroke onset (ICC 0.93), this variable was hampered with a substantial amount of missing values. Some variables related to treatment and examinations in hospital displayed low levels of agreement. This applies to heart rate monitoring (kappa 0.17/AC_1_ 0.46), swallowing test performed (kappa 0.19/AC_1_ 0.27) and mobilized out of bed within 24 h after admission (kappa 0.04/AC_1_ −0.11).

**Conclusion:**

A majority of the variables in The Norwegian Stroke Register have substantial to excellent reliability. The problem areas seem to be the lack of completeness in the time variable indicating stroke onset and poor reliability in some variables concerning examinations and treatment received in hospital.

**Electronic supplementary material:**

The online version of this article (doi:10.1186/s13104-015-1556-3) contains supplementary material, which is available to authorized users.

## Background

In many countries, there has been an increasing interest in medical quality registers as a tool for improving the quality of care and as sources of knowledge about diseases and of the health services. Consequently, a number of local, regional and national medical quality registers have been established. There seems to be agreement on the importance of such registers. However, there are challenges in obtaining valid and reliable data.

Few studies have investigated the validity of stroke registers [1], and these typically focus on calculating measures of completeness. Although some studies have investigated the reliability of selected key variables in registers [2, 3], we are aware of only one study assessing the reliability of *all* the variables in a stroke-specific register [4]. Reeves et al. found excellent inter-rater reliability for many variables, but several variables in need of improvement were also identified. These include stroke onset time, stroke team consultation, time of initial brain imaging and discharge destination.

Since 1 January 2012 all Norwegian hospitals are requested by law to report medical data on all hospitalized patients who fulfill the WHO criteria [5] for an acute stroke diagnosis to the Norwegian Stroke Register [6]. In the present study, we assessed the reliability of all the variables in the Norwegian Stroke Register by studying inter-rater reliability in a random sample of 111 patients.

## Methods

### The Norwegian Stroke Register

All hospitalized cases of acute stroke are to be reported to the Norwegian Stroke Register, irrespective of whether the patient was treated in a stroke unit or not. Data are initially registered on paper forms locally at the hospitals by dedicated and trained physicians and nurses, who subsequently enter data into the Stroke Register by use of a web based form. The completion of the registration process at the hospitals often takes place after the patient was discharged by look-up in the electronic medical records. As there are one or more registrars at each stroke unit or hospital, depending on the size of the unit, the reporting nurse or physician may or may not have been involved in the treatment of the patient.

The register contains person identifiable data on the patients’ functional status before the stroke, past medical history, the use of drugs prior to hospitalization and at discharge, clinical findings on admission to hospital, diagnostic procedures, treatment received during hospitalization and dates and times for stroke onset, admission to hospital and discharge. A user manual provides definitions of the variables and data entries [7].

### Data collection

The study population consists of patients hospitalized in one of four hospitals in Central Norway (St. Olav’s University Hospital, Levanger Hospital, Kristiansund Hospital and Ålesund Hospital). The four hospitals were chosen as they had reported data to the Stroke Register since 2004, and had by 2012 established well-functioning data collection routines. A total of 1253 patients were registered with an acute stroke diagnosis (ICD-10 codes I61, I63, I64) in the Norwegian Patient Register in the period of 1 April–31 December 2012 in the four hospitals under study. From these registrations, we selected 120 patients using a random number-generator.

An experienced nurse working at a stroke unit in one of the hospitals under study (St. Olav’s University Hospital) filled in the Norwegian Stroke Register paper forms for the 120 patients by a review of the patients’ medical records. She did not work at the stroke unit in 2012, and consequently did not perform the original registrations. The nurse was given access to all necessary information, including results of diagnostic tests and examinations and laboratory tests. The data collection was done during May–June 2014. The nurse did not receive any particular training; the only guidance she received was the Norwegian Stroke Register User Manual which is accessible to all registrars. The reason for this was that we wanted to mimic a “real world” situation as far as possible. The professional background of the nurse and the situation in which the registrations took place were considered typical to the actual registration procedures at hospitals around the country.

When extracting data from the Norwegian Stroke Register, 9 of the 120 cases were not found. Consequently, the sample size was reduced to 111 patients.

### Statistical analysis

The sample size was determined on the basis of recommended sample size calculations for the kappa statistic. The Goodness-of-fit approach by Donner and Eliasziw [8] states that based on alpha and beta error rates of 0.05 and 0.2, respectively, when testing for a statistical difference between moderate (0.40) and excellent (0.90) kappa values, sample size estimates range from 13 to 66. Our sample of 111 patients thus seems appropriate to detect generalizable estimates of inter-rater reliability.

For all nominal variables the inter-rater agreement is presented in terms of observed agreement, Cohen’s kappa and Gwet’s AC_1_ with 95 % confidence intervals (CI) [9–11]. Cases with missing values were excluded. For the three ordinal variables we used the quadratic weighted kappa and AC_2_, and the category “unknown” was excluded.

The kappa statistic is influenced by the trait prevalence and rater bias [12, 13]. In situations where a large proportion of the ratings are either positive or negative, the unbalanced prevalence of the trait will lead to a reduced kappa coefficient. In situations where there is a systematic difference between the two raters’ tendencies to make particular ratings, the kappa coefficient may be inflated. Gwet’s AC_1_ and AC_2_, however, is not affected by trait prevalence or rater bias [11].

When interpreting chance-corrected agreement, we use the criteria suggested by Landis and Koch [14], stating that a value between 0 and 0.20 implies slight agreement, 0.21–0.40 fair, 0.41–0.60 moderate, 0.61–0.80 substantial, and >0.80 excellent agreement. To aid interpretation of the agreement coefficients, cross tables for all presented variables are included in Additional file 1: Appendix S1.

Time variables were re-calculated as minutes past midnight when the corresponding date variable was the same for both raters (nurse and Stroke Register). We calculated the Intraclass Correlation Coefficient (ICC) using a two-way random effects consistency ANOVA model. ICC assesses agreement by comparing the variability of different ratings of the same subject to the total variation across all subjects [15]. The interpretation of the magnitude of ICC is similar to that of kappa; a coefficient of 0 means no agreement and 1 means full agreement. In addition to the ICC estimates, we calculated the mean and standard deviation of the differences between the raters.

When reporting results, we have strived to fulfil the recommendations in the Guidelines for Reporting Reliability and Agreement Studies by Kottner et al. [16]. We used IBM SPSS statistics 21 for building analysis files and for calculating ICC, and AgreeStat 2015.4 for calculating kappa, AC_1_ and AC_2_ statistics.

The study was approved by the Norwegian Directorate of Health and The Norwegian Data Protection Authority.

## Results

The sample of 111 patients consisted of 53.2 % men, with mean age 73.1 years (SD 14.9). In comparison, the total population in the Norwegian Stroke Register in 2012 consisted of 51.7 % men with mean age 74.1 years (no SD available) [17]. Additional file 2: Appendix S2 shows the number of hospitalizations with stroke and sample size for each of the four hospitals included in the study.

The agreement was substantial to excellent (kappa >0.60/AC_1_ >0.80) for most variables concerning functional status before stroke onset, past medical history and drug treatment prior to stroke onset (Table 1). For previous incidence of transient ischemic attack (TIA), the kappa of 0.54 indicated moderate agreement. However, an AC_1_ of 0.91 and an observed agreement of 91.9 % suggested that this variable had a skewed trait distribution and hence an artificially low kappa coefficient.

**Table 1:** Inter-rater reliability for medical history, functional status prior to stroke onset and pre-hospital data

	N	Obs.agr. (%)^a^	Kappa (95 % CI)	AC_1_ (95 % CI)
Past medical history				
Previous stroke	111	92.8	0.81 (0.69–0.94)	0.91 (0.85–0.97)
Myocardial infarction	111	96.4	0.87 (0.75–0.99)	0.95 (0.90–1.00)
Major cardiac interventions	111	92.8	0.66 (0.43–0.88)	0.92 (0.86–0.98)
Atrial fibrillation	111	89.2	0.71 (0.56–0.86)	0.87 (0.79–0.94)
Diabetes	109	97.2	0.92 (0.83–1.00)	0.96 (0.91–1.00)
TIA (transient ischemic attack)	111	91.9	0.54 (0.27–0.81)	0.91 (0.85–0.97)
Drug treatment prior to stroke onset				
Aspirin (ASA)	110	97.3	0.94 (0.87–1.00)	0.95 (0.89–1.00)
ADP receptor antagonist	110	98.2	0.74 (0.40–1.00)	0.98 (0.96–1.00)
Dipyridamole	110	95.5	0.60 (0.28–0.92)	0.95 (0.91–0.99)
Warfarin	111	98.2	0.93 (0.83–1.00)	0.98 (0.95–1.00)
Diuretics	111	86.5	0.60 (0.42–0.78)	0.84 (0.76–0.92)
ACE-inhibitor	110	98.2	0.91 (0.80–1.00)	0.98 (0.95–1.00)
A2-receptor blocker	110	92.7	0.70 (0.52–0.89)	0.92 (0.86–0.98)
Beta blocker	111	95.5	0.91 (0.82–0.99)	0.94 (0.90–0.99)
Calcium channel blocker	109	94.5	0.74 (0.54–0.94)	0.94 (0.90–0.99)
Statins	111	97.3	0.94 (0.87–1.00)	0.95 (0.90–1.00)
Other drugs against hypertension	111	72.0	0.48 (0.34–0.61)	0.63 (0.51–0.74)
Functional status prior to stroke				
Housing^b^	110	98.2	0.88 (0.80–0.96)	0.97 (0.94–0.99)
Functional mobility^b^	104	98.1	0.83 (0.67–0.98)	0.98 (0.96–0.99)
Marital status	111	75.7	0.62 (0.52–0.72)	0.69 (0.58–0.80)
Living situation	106	91.0	0.84 (0.75–0.93)	0.89 (0.82–0.96)
Toilet hygiene	111	88.3	0.44 (0.24–0.65)	0.87 (0.80–0.94)
Dressing	111	87.4	0.55 (0.37–0.73)	0.85 (0.78–0.93)
On stroke onset				
Patient woke with stroke symptoms?	108	62.0	0.28 (0.11–0.44)	0.49 (0.35–0.62)
Place of stroke onset	110	96.4	0.58 (0.21–0.96)	0.96 (0.92–1.00)

^a^Observed agreement calculated as concordant answers divided by N

^b^Ordinal data, estimate based on Cohen’s quadratic weighted kappa and Gwet’s AC_2_

The variable *patient woke with stroke symptoms* had a kappa coefficient of 0.28 and an AC_1_ of 0.49, indicating fair to moderate agreement. For the variable *place of stroke onset* a kappa of 0.58 was balanced by an AC_1_ of 0.96 and an observed agreement of 96.4 %, thus the reliability was considered to be good for this variable.

Variables describing FAST-symptoms (hemiparesis and speech problems) showed moderate to substantial agreement (kappa 0.52–0.64/AC_1_ 0.62–0.73), while agreement for other focal symptoms were moderate (kappa 0.37/AC_1_ 0.46). The variable describing side of symptom manifestation showed substantial agreement (kappa 0.69/AC_1_ 0.73). *Level of consciousness* is an ordinal variable displaying substantial to excellent agreement (kappa 0.66/AC_2_ 0.93) (Table 2).

**Table 2:** Inter-rater reliability for symptoms, diagnostic procedures, treatment and stroke subtype

	N	Obs.agr. (%)^a^	Kappa (95 % CI)	AC_1_ (95 % CI)
Symptoms at admission				
Hemiparesis face	108	79.6	0.64 (0.51–0.77)	0.72 (0.61–0.83)
Hemiparesis legs	110	73.6	0.54 (0.41–0.68)	0.63 (0.51–0.75)
Hemiparesis arms	109	80.0	0.62 (0.49–0.75)	0.73 (0.62–0.83)
Speech problems	107	72.9	0.52 (0.37–0.66)	0.62 (0.50–0.75)
Other focal symptoms	111	62.2	0.37 (0.22–0.53)	0.46 (0.33–0.59)
Symptoms at right/left side	111	79.3	0.69 (0.58–0.79)	0.73 (0.64–0.84)
Level of consciousness^b^	109	95.8	0.66 (0.47–0.85)	0.93 (0.89–0.98)
Diagnostic imaging and examinations				
CT/MRI <24 h after admission	110	97.3	0.69 (0.39–0.99)	0.97 (0.94–1.00)
Imaging of the stroke	109	87.2	0.77 (0.66–0.87)	0.84 (0.76–0.92)
Imaging of intracranial vessels	110	91.0	0.56 (0.33–0.79)	0.90 (0.85–0.96)
Imaging of extracranial vessels	110	83.6	0.71 (0.59–0.83)	0.82 (0.74–0.90)
Imaging of the heart	111	89.2	0.68 (0.52–0.85)	0.88 (0.82–0.95)
Heart rate monitoring	111	52.2	0.17 (0.03–0.31)	0.46 (0.35–0.57)
Treatment and diagnosis				
Anticoagulants as treatment	111	88.3	0.34 (0.04–0.60)	0.87 (0.80–0.94)
Anticoagulants as prophylaxis	111	86.5	0.56 (0.36–0.76)	0.84 (0.76–0.92)
Swallowing test performed	111	48.6	0.19 (0.09–0.30)	0.27 (0.12–0.43)
Mobilized out of bed <24 h	110	21.8	0.04 (0.00–0.09)	−0.11 (0.00–0.01)
Stroke subtype	111	99.1	0.97 (0.90–1.00)	0.99 (0.97–1.00)

^a^Observed agreement calculated as concordant answers divided by N

^b^Ordinal data, estimate based on Cohen’s quadratic weighted kappa and Gwet’s AC_2_

For variables concerning diagnostic imaging there was substantial to excellent agreement (kappa > 0.56/AC_1_ > 0.82). However, the variable *heart rate monitoring* had slight to moderate agreement (kappa 0.17/AC_1_ 0.46).

The variable *mobilized out of bed within 24* *h after admission* had no better agreement than by chance (kappa 0.04/AC_1_ −0.11), and the variable *swallowing test performed* showed only slight to fair agreement (kappa 0.19/AC_1_ 0.27).

For the stroke subtype variable, there was close to perfect agreement (kappa 0.97/AC_1_ 0.99).

All response options in the variable *discharge destination* indicated substantial agreement (kappa 0.69/AC_1_ 0.72) (Table 3). Drug treatment at discharge seemed to be varying in the level of agreement between the different types of drug, from a kappa of 0.25/AC_1_ of 0.65 for Dipyridamole as the least reliable variable to kappa and AC1 > 0.90 for ADP receptor antagonist, ACE inhibitor, A2 receptor blocker and statins as the most reliable variables.

**Table 3:** Inter-rater reliability for discharge data

	N	Obs.agr. (%)^a^	Kappa (95 % CI)	AC_1_ (95 % CI)
Discharge destination				
Discharge destination	111	74.8	0.69 (0.60–0.79)	0.72 (0.63–0.81)
Drug treatment at discharge				
Aspirin (ASA)	110	73.6	0.51 (0.38–0.64)	0.65 (0.54–0.76)
ADP receptor antagonist	110	99.1	0.94 (0.83–1.00)	0.99 (0.97–1.00)
Dipyridamole	110	71.0	0.25 (0.10–0.41)	0.65 (0.53–0.76)
Warfarin	110	96.4	0.85 (0.70–0.99)	0.96 (0.92–0.99)
Diuretics	110	88.2	0.60 (0.40–0.80)	0.86 (0.79–0.94)
ACE-inhibitor	110	99.1	0.96 (0.90–1.00)	0.99 (0.97–1.00)
A2-receptor blocker	110	93.6	0.75 (0.58–0.93)	0.93 (0.87–0.98)
Beta blocker	110	94.5	0.87 (0.77–0.97)	0.93 (0.88–0.99)
Calcium channel blocker	109	96.3	0.83 (0.66–0.99)	0.96 (0.92–0.99)
Statins	110	96.4	0.93 (0.86–1.00)	0.95 (0.93–1.00)

^a^Observed agreement calculated as concordant answers divided by N

There was a substantial amount of missing values in date (24.3 %) and time (58.6 %) of stroke onset in the data recorded by the nurse and in time of stroke onset in the Stroke Register (40.5 %). Time of hospital admission was missing in 13.5 % of the cases for the nurse and 2.7 % of the cases for the Stroke Register. As a consequence, only 42 and 89 cases out of 111 were included in the calculation of ICC for stroke onset time and hospital admittance time, respectively (Table 4).

**Table 4:** Intraclass correlation coefficient for two time variables

Variable	N	ICC (95 % CI)	Mean diff.^b^	SD^c^
Stroke onset time (min)^a^	42	0.93 (0.88–0.96)	26.38	121.60
Hospital admission time (min)^a^	89	0.98 (0.97–0.99)	0.45	64.50

^a^Calculated as minutes past midnight

^b^Mean difference in minutes calculated as nurse minus Stroke Register

^c^Standard deviation of the difference

Both time variables had excellent agreement (ICC 0.93–0.98) where there was a value recorded. However, the mean difference and variance between the raters were greater for stroke onset time than for hospital admission time, as was the level of incompleteness.

## Discussion

Most of the variables in the Norwegian Stroke Register appeared to have substantial to excellent reliability, including many of the variables related to past medical history, functional status before the stroke, discharge destination, stroke subtype and drug treatment prior to the stroke. Variables related to focal symptoms and speech problems at admittance to hospital showed moderate agreement, while the variable describing whether the patient woke with symptoms indicated slight to moderate agreement. Furthermore, reliability was low for variables related to several examinations and clinical findings during hospitalization, such as monitoring heart rate, test of swallowing function and whether the patient was mobilized out of bed <24 h after admission.

When interpreting the results, for variables with substantial discrepancy between the kappa and AC_1_ coefficients, the variable was considered reliable where kappa was low and AC_1_ and observed agreement was high. In these cases the kappa coefficient was considered artificially low due to skewed trait prevalence. Distribution of trait prevalence for all variables is shown in Additional file 1: Appendix S1.

Time of stroke onset is a particularly important variable, as it is essential in determining eligibility for thrombolytic therapy. In this study, time of stroke onset and admission to hospital indicated excellent agreement where such time was in fact registered. However, there was a large proportion of missing values in these data. The data collection procedures in this study appear to have had some impact on the data completeness. For the nurse, using paper forms to fill in the data, there was a substantial amount of missing values in both date and time of stroke onset and in time of hospital admission. In the Stroke Register, on the other hand, time of stroke onset was the only date/time-variable which was hampered with a high degree of missingness. A likely explanation is that the content in the Norwegian Stroke Register is collected via an online electronic form where the fields for stroke onset date and hospital admission date are mandatory fields. The time variables, however, are not mandatory.

Other studies have found a similar high degree of missingness when recording time of stroke onset [4, 18, 19]. Proposed suggestions to correct this weakness in the data have ranged from developing a real-time data collection system for recording stroke onset time, to the use of standardized time windows instead of the actual hour/minute of the onset [20]. Although this study indicated a high degree of reliability for the date and time variables, the results suggests that steps needs to be taken to ensure more complete recordings of time of stroke onset in the Norwegian Stroke Register.

The registration of stroke symptoms displayed moderate levels of agreement. One can expect to see some variation in level of agreement for these types of variables, as they are to some extent based on subjective assessments and thereby inherently difficult to classify in a consistent manner [21]. A study investigating inter-rater reliability for clinical assessment of stroke found no better agreement between clinicians assessing focal symptoms in the same patients than what we found in the present study [22].

Another problematic variable was the recording of heart rate monitoring. Almost all of the registrations in this variable fell within two categories; ECG alone or a combination of several modalities (ECG, telemetry, Holter monitoring). However, the poor level of agreement suggests that a clarification of the response categories in this variable can be useful.

The Norwegian Stroke Register’s User Manual does not provide any criteria for recording whether swallowing function was tested. Given the poor reliability of this variable, there seems to be some confusion as to the definition of such a test. For the variable *mobilized out of bed* <24 h *after admission* the raw data showed that the main difference is that the nurse chose the category “unknown” to a larger extent than the stroke register. This could indicate that for this variable, it is difficult to find clear information in the medical records. In further quality enhancement work, the Stroke Register should look into the possibility of better definitions or explanations of these variables.

This study has several limitations. First, differences in data collection methods between the nurse and the Stroke Register may have had an impact on the results. This is particularly applicable for the date variables, as these are mandatory fields in the electronic web-based form of the Norwegian Stroke Register, while the nurse using paper forms did not have any validation rules affecting her registrations. Second, the nurse recorded data for four different hospitals, while the stroke register contains data collected by one registrar per hospital. Third, the sample size is not sufficient to perform analyses at the level of each hospital. Finally, 2012 was the first year after the stroke register was established as a national register and most hospitals had limited experience with documentation in the medical records and registration in the register. Hence the results might improve in the future.

Additionally, when making inferences based on reliability studies, there is an inherent challenge in determining whether discrepancies between the two raters’ registrations are due to factors related to the quality of the hospital medical records, the quality of variables in the register or the quality of the registration work by the raters.

## Conclusion

A majority of the variables in the Norwegian Stroke Register have substantial to excellent reliability. The problem areas seem to be the level of incompleteness in the time variable related to stroke onset and poor reliability in some variables concerning examinations, clinical findings and treatment during hospitalization. The study points to ambiguous definitions of variables or response categories as well as difficulties in finding or interpreting information in medical records as possible explanations to the discrepancies. Steps should be taken to improve the completeness and reliability of the variables in question.

## Additional files

10.1186/s13104-015-1556-3 Contingency tables of all variables.
10.1186/s13104-015-1556-3 Number of hospitalizations and sample size for each of the four hospitals included in the study.

## Abbreviations

CI: Confidence interval
ECG: Electrocardiography
FAST: Face, arm, speech, time
ICC: Intraclass correlation coefficient
ICD-10: International Classification of Diseases version 10
SD: Standard deviation
TIA: Transient ischemic attack
WHO: World Health Organization
