# Supplementary material for: Inter-rater reliability of a national acute stroke register
Source: BMC Res Notes. 2015 Oct 19;8:584. doi: 10.1186/s13104-015-1556-3 (PMC4617717; doi:10.1186/s13104-015-1556-3)
Supplement: Supplementary file 2 — 10.1186/s13104-015-1556-3 Number of hospitalizations and sample size for each of the four hospitals included in the study. [file 13104_2015_1556_MOESM2_ESM.docx]

Appendix S2: Inter-rater Reliability of a National Acute Stroke Register

Hospitalizations for acute stroke in the study period per hospital, sample size per hospital and missingness in the variable stroke onset time.

|  | St. Olav’s Uni. Hospital | Levanger Hospital | Kristiansund Hospital | Ålesund Hospital | Total |
| --- | --- | --- | --- | --- | --- |
| Total no. of hospitalizations with a stroke diagnosis, April 1- December 31 2012, n (%) | 727 (58.0%) | 218 (17.4%) | 116 (9.3%) | 192 (15.3%) | 1253 (100%) |
| Hospitalizations in the sample, n (%) | 35 (31.5%) | 1 (0.9%) | 46 (41.4%) | 29 (26.1%) | 111 (100%) |
| Missing stroke onset time – nurse, n (%) | 21 (32.3%) | 1 (1.5%) | 26 (40.0%) | 17 (26.2%) | 65 (100%) |
| Missing stroke onset time- Stroke Register, n (%) | 14 (31.1%) | 1 (2.2%) | 17 (37.8%) | 13 (28.9%) | 45 (100%) |
